# The circadian–melatonin axis in breast cancer metastasis: molecular mechanisms, organ-specific metastatic niches, and therapeutic opportunities

**DOI:** 10.3389/fendo.2026.1941609

**Published:** 2026-09-03

**Authors:** Ching-Hsiang Wang, Kian-Hwee Chong, Ching-Feng Cheng, Yao-Jen Chang, Kuo-Wang Tsai

**Affiliations:** 1 Division of Gastroenterology, Department of Internal Medicine, Taoyuan Armed Forces General Hospital, Taoyuan, Taiwan; 2 Division of General Surgery, Department of Surgery, Taipei Tzu Chi Hospital, Buddhist Tzu Chi Medical Foundation, New Taipei, Taiwan; 3 School of Medicine, Tzu Chi University, Hualien, Taiwan; 4 Department of Pediatrics, Taipei Tzu Chi Hospital, Buddhist Tzu Chi Medical Foundation, New Taipei City, Taiwan; 5 Department of Research, Taipei Tzu Chi Hospital, Buddhist Tzu Chi Medical Foundation, New Taipei City, Taiwan; 6 Department of Nursing, Cardinal Tien Junior College of Healthcare and Management, New Taipei City, Taiwan; 7 Department of Post-Baccalaureate Nursing, Tzu Chi University, Hualien, Taiwan

**Keywords:** breast cancer, circadian rhythm, melatonin, metastasis, MT1 receptor

## Abstract

Breast cancer metastasis remains the leading cause of disease-related mortality despite major advances in systemic therapy. Increasing evidence indicates that the circadian–melatonin axis may influence metastatic progression by coordinating endocrine signaling, metabolism, immune responses, and tumor cell plasticity. In this review, we summarize current epidemiological, mechanistic, and translational evidence linking circadian disruption and melatonin to breast cancer metastasis. We discuss how core circadian clock genes and melatonin signaling may influence epithelial–mesenchymal transition, cancer stemness, circulating tumor cell dissemination, tumor metabolism, and immune remodeling. We further highlight the emerging potential roles of melatonin in shaping adipocyte–tumor crosstalk and organ-specific metastatic niches, including bone, lung, liver, and brain, where tissue-specific interactions may influence metastatic colonization. Finally, we discuss the clinical potential of circadian chronotherapy, adjuvant melatonin, and circadian biomarker-guided precision medicine. Although substantial experimental evidence supports the anti-metastatic properties of the circadian–melatonin axis, clinical translation remains limited by context-dependent biological effects and the lack of chronobiology-informed trials. Future studies integrating circadian biomarkers with optimized treatment timing and modern systemic therapies may facilitate precision strategies for preventing and treating metastatic breast cancer.

## 1 Introduction

Breast cancer accounts for approximately one in eight cancers diagnosed globally and, despite substantial gains in early detection and systemic therapy, remains the leading cause of cancer death in women (1). The natural history of the disease is dominated by distant metastasis: five-year survival exceeds 90% for localized disease but collapses to below 30% once metastatic dissemination has occurred, with organ-specific outcomes ranging from a comparatively indolent course in bone-dominant disease to a median survival of less than one year in brain-only recurrence (2).

The metastatic cascade comprises local invasion, intravasation, hematogenous dissemination via circulating tumor cells (CTCs), arrest and extravasation, and metastatic colonization, and has traditionally been viewed as a linear, temporally ordered process. This traditional view has since been challenged. Studies in mouse models and, importantly, in patients with metastatic breast cancer have demonstrated that CTC shedding occurs predominantly during the rest phase and that rest-phase CTCs exhibit greater proliferative and metastatic potential than those released during the active phase (3). Together with epidemiological and mechanistic evidence linking circadian disruption and night-shift work to breast cancer risk, these findings have established the concept that the circadian timing system is not merely a regulator of metabolism and sleep but also a key biological determinant of tumor progression (4–6).

The pineal indoleamine melatonin (*N*-acetyl-5-methoxytryptamine) is the principal hormonal signature of biological night. Its production is directly suppressed by ocular exposure to light. Melatonin acts through two high-affinity Gi-protein-coupled receptors, MT1 (*MTNR1A*) and MT2 (*MTNR1B*), and through receptor-independent antioxidant activity. Physiological nocturnal melatonin concentrations suppress tumor uptake of linoleic acid, downregulate aerobic glycolysis, and inhibit growth of ER-positive and ER-negative xenografts perfused *in situ* (7, 8). Melatonin suppresses several biological processes implicated in metastatic progression, including RANKL-driven osteoclastogenesis and pro-angiogenic signaling, supporting its role as a potential endogenous suppressor of metastasis (9, 10).

However, the role of melatonin in metastatic progression appears to be more complex than initially appreciated. Diamantopoulou et al. demonstrated that exogenous melatonin administered before the rest phase increased CTC shedding in tumor-bearing mice (3), suggesting that the biological effects of melatonin depend critically on its timing relative to both the host circadian rhythm and the intrinsic circadian clock of tumor cells. Collectively, these findings indicate that the circadian–melatonin axis should be viewed as a dynamic, temporally regulated signaling network rather than as the action of a single molecule with a uniform biological effect.

In this Review, we synthesize epidemiological, mechanistic, and translational evidence supporting the role of the circadian–melatonin axis in breast cancer metastasis, with particular emphasis on organ-specific metastatic colonization and the local tissue microenvironment. Finally, we discuss the translational potential of chronotherapy and melatonin as an adjuvant therapy.

## 2 The circadian timing system and pineal melatonin: a primer

**Figure 1:**
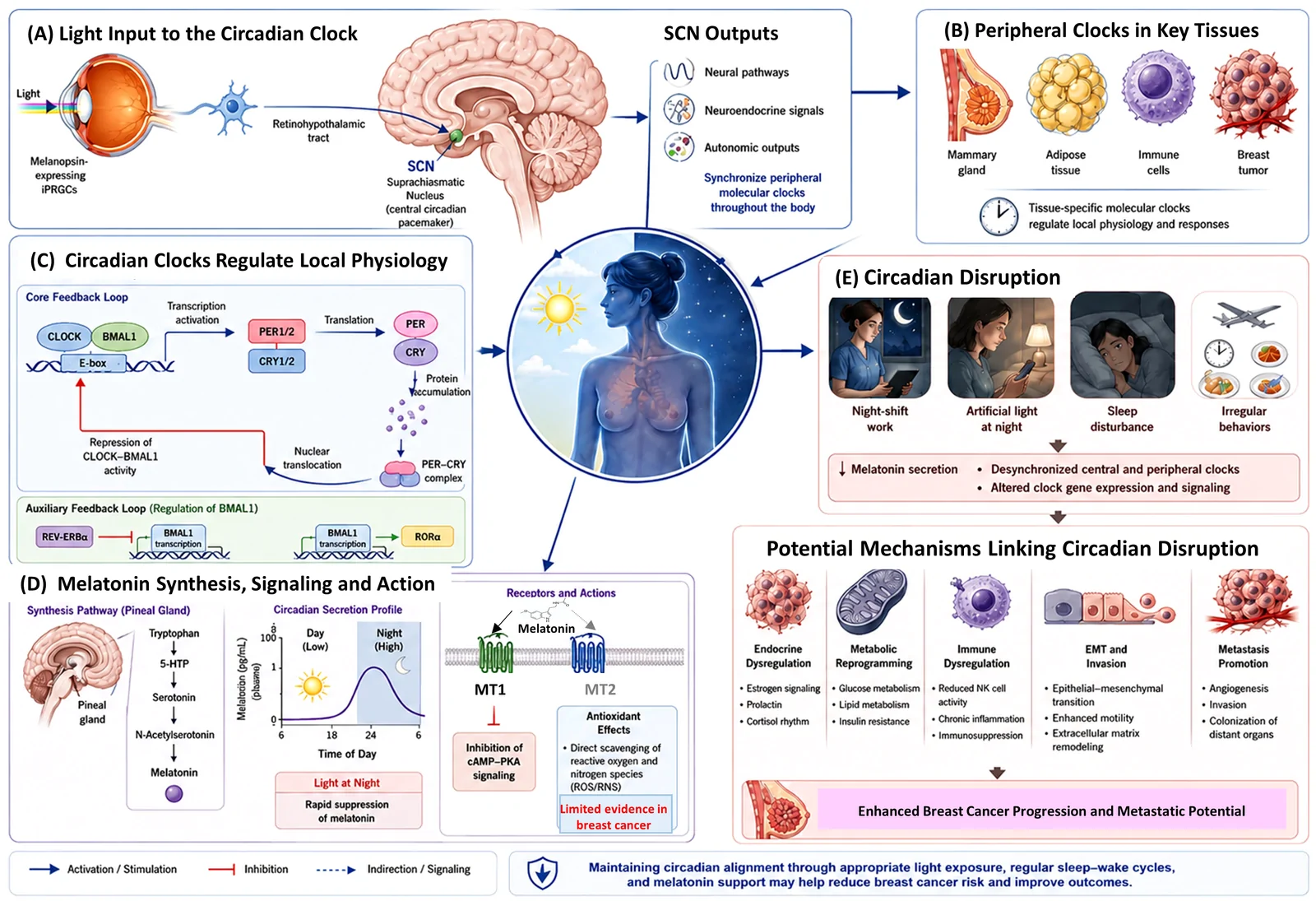
Overview of the circadian timing system and melatonin signaling. **(A)** Light entrains the SCN through ipRGCs. **(B)** The SCN synchronizes peripheral clocks. **(C)** The molecular clock consists of interconnected transcription–translation feedback loops. **(D)** Melatonin is synthesized in the pineal gland and signals primarily through MT1. **(E)** Circadian disruption suppresses nocturnal melatonin and may contribute to breast cancer progression and metastasis.

As summarized in **Figure 1**, environmental light entrains the central circadian clock through melanopsin-expressing intrinsically photosensitive retinal ganglion cells (ipRGCs), enabling the SCN to synchronize peripheral clocks throughout the body.

### 2.1 The circadian clock: from the SCN to peripheral and tumor clocks

Nearly every mammalian cell contains an intrinsic molecular clock based on a transcription–translation feedback loop. In the core loop, the CLOCK–BMAL1 heterodimer activates the transcription of PER and CRY genes through E-box elements. As PER and CRY proteins accumulate, they translocate back to the nucleus and inhibit CLOCK–BMAL1 activity, thereby generating a self-sustaining ~24-hour oscillation. A secondary feedback loop involving REV-ERBα and RORα further stabilizes BMAL1 rhythmicity (**Figure 1C****) (**11, 12).

At the organismal level, circadian rhythms are coordinated by the suprachiasmatic nucleus (SCN), the master pacemaker located in the anterior hypothalamus. The SCN receives environmental light signals through melanopsin-expressing ipRGCs and synchronizes peripheral clocks throughout the body, including those in the liver, adipose tissue, breast epithelium, immune cells, and tumor cells (**Figures 1A, B**) (11, 12). Importantly, accumulating evidence indicates that breast tumors retain functional circadian clocks. Multi-omic analyses of human breast cancers demonstrated that circadian gene expression is largely preserved in luminal A tumors, which exhibit a relatively more organized intrinsic molecular clock than other breast cancer subtypes. Experimental disruption of the intrinsic tumor clock promoted epithelial–mesenchymal transition (EMT) and increased metastatic potential, indicating that circadian regulation extends beyond systemic physiology to directly influence metastatic progression (13). Importantly, preservation of intrinsic clock organization should not be equated with increased global circadian rhythm strength (CYCLOPS magnitude). While luminal A tumors generally retain a relatively organized molecular clock, individual tumors exhibit substantial heterogeneity in global circadian rhythm strength. Recent transcriptomic analyses further demonstrated that tumors with higher CYCLOPS magnitude displayed enhanced rhythmicity of EMT-associated pathways and poorer clinical outcomes. These findings suggest that intrinsic clock organization and global rhythm strength represent distinct biological dimensions of the tumor circadian system and should not be interpreted interchangeably (13).

### 2.2 Melatonin biosynthesis, secretion, and signaling

Melatonin is synthesized predominantly in the pineal gland through the sequential conversion of tryptophan to serotonin, N-acetylserotonin, and finally melatonin, with arylalkylamine N-acetyltransferase (AANAT) serving as the rate-limiting enzyme. Its secretion is tightly regulated by the circadian clock, resulting in a pronounced nocturnal rise that is rapidly suppressed by nocturnal light exposure, particularly blue-enriched light (14). Melatonin exerts its biological effects primarily through two high-affinity G protein-coupled receptors, MT1 (MTNR1A) and MT2 (MTNR1B), which couple to Gi/o proteins and inhibit adenylyl cyclase activity, thereby reducing intracellular cyclic AMP (cAMP) levels and downstream protein kinase A (PKA) signaling (**Figure 1D**). In addition to these membrane receptors, melatonin has been reported to interact with quinone reductase 2 (formerly designated MT3), although the physiological significance of this interaction remains incompletely understood. Beyond receptor-mediated signaling, melatonin and its metabolites, including N¹-acetyl-N²-formyl-5-methoxykynuramine (AFMK) and N¹-acetyl-5-methoxykynuramine (AMK), directly scavenge reactive oxygen and nitrogen species, contributing to its potent antioxidant activity. Melatonin has also been proposed to regulate gene transcription through interactions with retinoic acid receptor-related orphan receptors (ROR/RZR), although this mechanism remains controversial and has yet to be conclusively established (15, 16). Owing to its lipophilic nature, melatonin readily crosses biological barriers, including the blood–brain barrier, allowing widespread distribution throughout the body, a property that contributes to its growing therapeutic interest in oncology (16, 17).

## 3 Circadian disruption and breast cancer: epidemiological evidence

The IARC first classified shift work involving circadian disruption as probably carcinogenic to humans (Group 2A) in 2007 and reaffirmed this conclusion in Volume 124 (2020), with breast cancer representing one of the strongest cancer associations (4, 18). The 2019 IARC evaluation was further supported by a 2024 review, which concluded that persistent night-shift work, particularly of long duration or high frequency, continues to be associated with a modest increase in breast cancer risk despite persistent methodological heterogeneity across epidemiological studies (19). A recent systematic review and meta-analysis of 21 studies (n = 586,890) published between 2012 and 2023 found no consistent overall association between night-shift work and breast cancer risk. Although significant associations were observed in cohort studies, substantial heterogeneity across study designs and exposure assessment precluded definitive conclusions (20). Circadian rhythm–disrupting behaviors, particularly prolonged sleep duration, poor sleep quality, and shorter nightly fasting duration, have been associated with poorer breast cancer outcomes in a systematic review of breast cancer survivors (21). Collectively, these findings suggest that circadian disruption is clinically relevant to breast cancer, although the magnitude and consistency of epidemiological associations remain modest and heterogeneous (**Figure 1E**).

## 4 Molecular mechanisms of the circadian–melatonin axis in breast cancer

### 4.1 Key circadian clock genes as tumor suppressors and oncogenes

**Figure 2:**
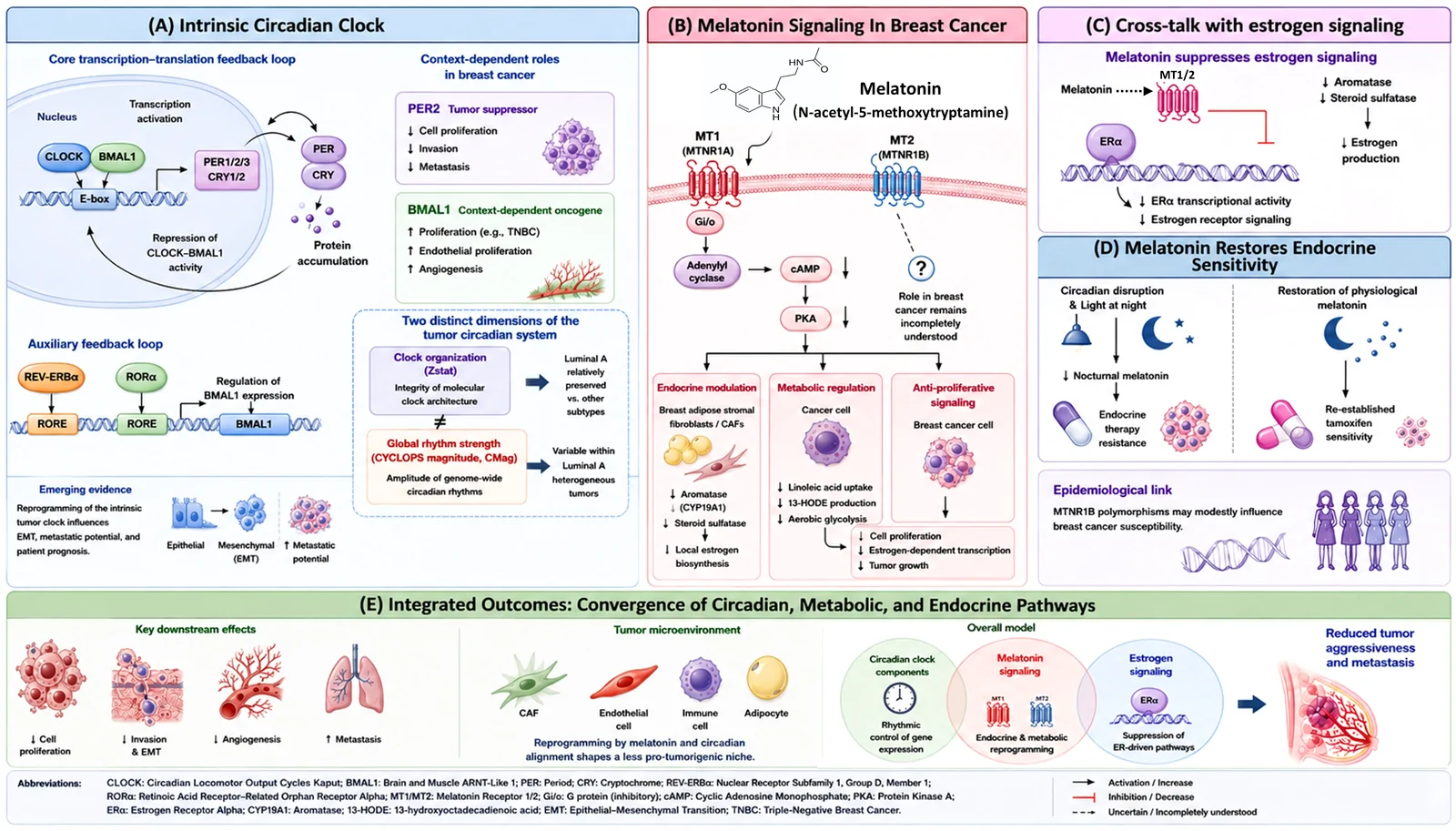
Molecular mechanisms of the circadian–melatonin axis in breast cancer. **(A)** Core circadian clock components regulate breast cancer progression in a context-dependent manner. **(B)** Melatonin signaling is mediated predominantly through MT1, leading to endocrine, metabolic, and anti-proliferative effects. **(C)** Melatonin suppresses estrogen signaling by inhibiting ERα activity and local estrogen biosynthesis. **(D)** Restoration of physiological melatonin may improve endocrine sensitivity under conditions of circadian disruption. **(E)** Circadian clock components, melatonin signaling, and estrogen pathways converge to regulate tumor metabolism, the tumor microenvironment, and breast cancer progression.

PER2 is one of the best-characterized circadian tumor suppressors in breast cancer. Early functional studies demonstrated that restoration of PER2 expression suppresses breast cancer cell proliferation and invasion (22, 23). BMAL1 is required for endothelial cell-cycle progression and physiological angiogenesis, highlighting an important role for circadian signaling in vascular remodeling (24). BMAL1 has been reported to exhibit oncogenic functions in several breast cancer models. Silencing BMAL1 in both *in vitro* and *in vivo* models significantly enhances apoptosis while suppressing the proliferation of triple-negative breast cancer (TNBC) cells (25). The functions of CLOCK and CRY family members appear to be highly context-dependent, varying across molecular subtypes, tumor microenvironments, and cellular contexts, underscoring the complexity of circadian regulation in breast cancer biology (26).More recently, transcriptomic analyses of human breast cancers demonstrated that circadian gene-expression rhythms are partially preserved but undergo substantial reprogramming in luminal A tumors. Importantly, preservation of intrinsic clock organization does not necessarily correspond to increased global circadian rhythm strength (CYCLOPS magnitude, CMag). Within luminal A tumors, those exhibiting higher CMag displayed enhanced rhythmicity of EMT-associated pathways, greater metastatic potential, and poorer five-year survival, suggesting that global rhythm strength reflects a distinct dimension of tumor circadian biology beyond intrinsic clock organization (13). Collectively, these observations indicate that individual clock components cannot be uniformly classified as tumor suppressors or oncogenes, because their functions depend on molecular subtype, cellular state, and different aspects of circadian regulation, including both clock integrity and global rhythm strength (**Figure 2A**).

### 4.2 Melatonin receptor signaling in breast cancer

MT1 is expressed in human breast cancers and is more abundantly expressed in ER-positive tumors than in ER-negative disease (27, 28). Upon melatonin binding, MT1 activates Gi proteins, suppressing adenylyl cyclase activity and reducing intracellular cAMP levels, thereby attenuating cAMP/PKA signaling (29). Activation of MT1 suppresses breast cancer cell proliferation and mediates anti-estrogenic effects (29). In addition, melatonin suppresses aromatase (CYP19A1) expression in breast adipose stromal fibroblasts and cancer-associated fibroblasts, thereby reducing local estrogen biosynthesis within the tumor microenvironment (30, 31). The MT1-mediated melatonin signaling pathway also regulates breast cancer metabolism by suppressing linoleic acid uptake and its conversion to the mitogenic lipid 13-hydroxyoctadecadienoic acid (13-HODE). In tissue-isolated human breast cancer xenografts perfused *in situ*, physiological nocturnal melatonin markedly reduced linoleic acid uptake, 13-HODE production, aerobic glycolysis, and tumor growth (7). These inhibitory effects were abolished by the non-selective melatonin receptor antagonist S20928, demonstrating that melatonin regulates tumor metabolism through a receptor-mediated signaling pathway (**Figure 2B**) (32, 33).

Although both MT1 and MT2 are membrane melatonin receptors, functional studies in breast cancer have predominantly implicated MT1. MT2 expression is absent or expressed at very low levels in most commonly used breast cancer cell lines, and direct evidence supporting an independent role for MT2 in breast cancer progression remains limited (29). Despite the limited functional evidence for MT2 in breast cancer, several population-based genetic association studies have investigated whether MTNR1B polymorphisms influence disease susceptibility. To date, several epidemiological studies have suggested that common genetic variants in MTNR1B may contribute to breast cancer susceptibility (34, 35). However, these associations are generally modest and vary according to the specific polymorphism and population studied. Moreover, the reported findings have not been consistently replicated across different cohorts, suggesting that additional large-scale validation studies are warranted (34, 36). Collectively, current evidence indicates that MT1 is the principal functional melatonin receptor in breast cancer, whereas the biological significance of MT2 remains largely unresolved despite emerging genetic association studies.

### 4.3 Cross-talk with estrogen signaling

Melatonin exerts anti-estrogenic effects through multiple mechanisms. It suppresses ERα-dependent transcription while inhibiting aromatase and steroid sulfatase, thereby reducing local estrogen biosynthesis within the breast tumor microenvironment (**Figure 2C**) (37–39). Emerging evidence suggests that melatonin may improve the efficacy of endocrine therapy through modulation of estrogen signaling and circadian regulation, although further clinical validation is still required (37). In a landmark preclinical study, Dauchy et al. demonstrated that dim-light-at-night–induced suppression of nocturnal melatonin rendered ER-positive MCF-7 xenografts resistant to tamoxifen, whereas restoration of the nocturnal melatonin signal re-established tamoxifen sensitivity (**Figure 2D**) (8). These findings provide a strong preclinical rationale for investigating appropriately timed nocturnal melatonin as an adjunct to endocrine therapy in patients with ER-positive breast cancer. Clinical studies have shown that melatonin is generally well tolerated and may improve sleep quality, fatigue, and treatment-related symptoms; however, robust evidence demonstrating improved endocrine therapy efficacy or survival outcomes in ER-positive breast cancer remains lacking (37). Collectively, these interconnected circadian, metabolic, and endocrine pathways converge to regulate breast cancer progression and are summarized in **Figure 2E**.

## 5 Melatonin and the metastatic cascade

### 5.1 Local invasion, extracellular matrix remodeling

**Figure 3:**
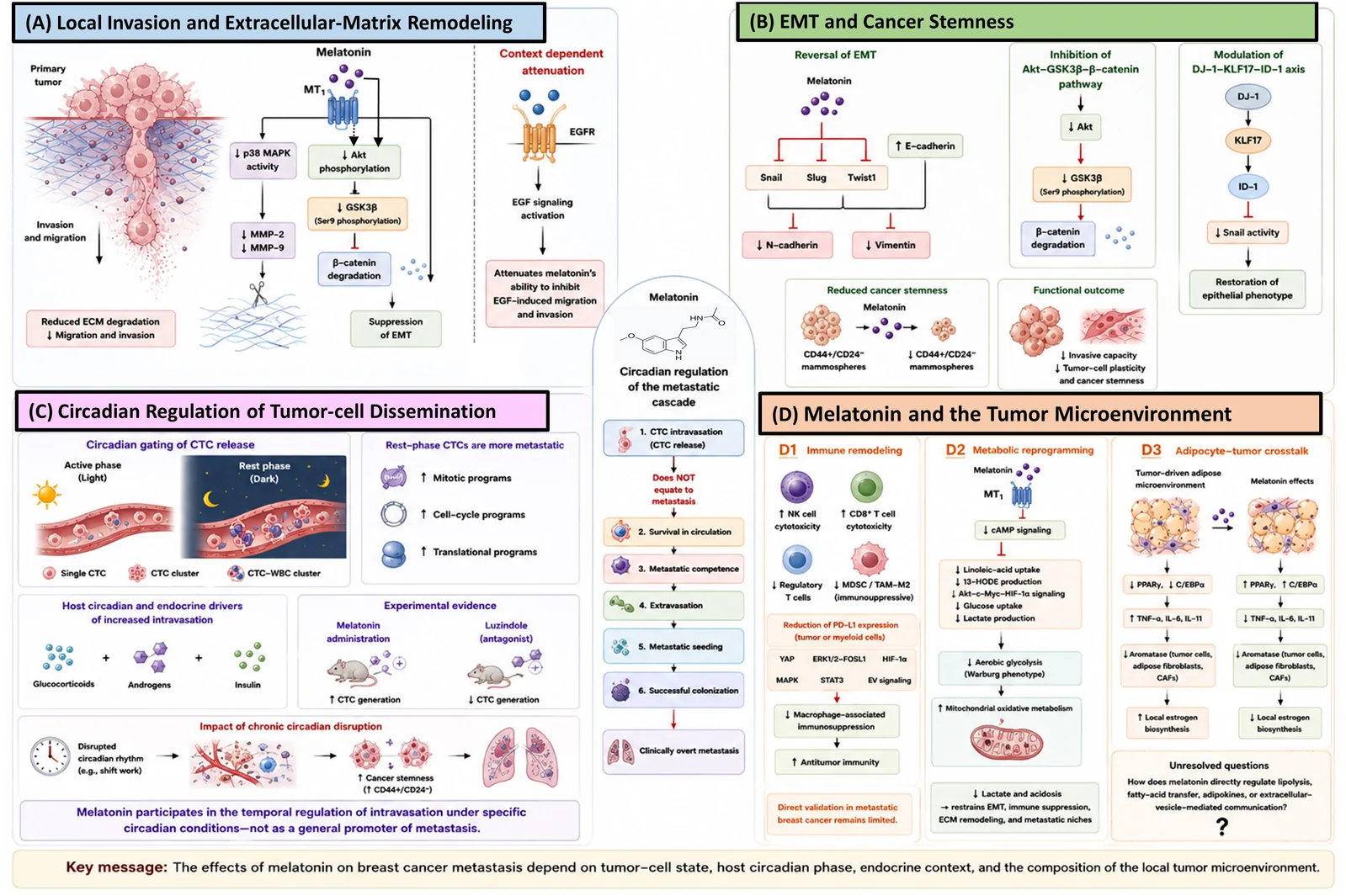
Melatonin regulates the metastatic cascade and tumor microenvironment in breast cancer. **(A)** Melatonin suppresses local invasion and extracellular-matrix remodeling by inhibiting EMT- and migration-related signaling pathways. **(B)** Melatonin reverses EMT and reduces cancer stemness through modulation of multiple signaling pathways. **(C)** Circadian rhythms and melatonin influence the temporal regulation of circulating tumor-cell dissemination in a context-dependent manner. **(D)** Melatonin remodels the tumor microenvironment through immune regulation, metabolic reprogramming, and adipocyte–tumor crosstalk, thereby contributing to reduced metastatic progression.

Melatonin suppresses breast cancer cell migration and invasion through multiple molecular mechanisms. In invasive MCF-7-derived breast cancer models, melatonin inhibits MT1-dependent p38 MAPK signaling, leading to reduced expression and activity of MMP-2 and MMP-9 (40). A recent study similarly reported that melatonin reduced MMP2 and MMP9 expression and suppressed migration and invasion in breast cancer cells (41). In addition, melatonin suppresses Akt phosphorylation, activates GSK3β, promotes β-catenin degradation, and inhibits epithelial–mesenchymal transition (EMT), thereby reducing metastatic potential (42). Interestingly, not all studies have demonstrated anti-metastatic effects of melatonin. In a recent study, melatonin failed to suppress EGF-induced migration and invasion, suggesting that activation of EGF signaling may attenuate the anti-tumor activity of melatonin under certain conditions (**Figure 3A**) (43).

### 5.2 Epithelial–mesenchymal transition and cancer stemness

EMT is a master developmental program through which epithelial breast cancer cells lose apical–basal polarity, downregulate E-cadherin, and acquire mesenchymal characteristics that facilitate invasion and metastatic dissemination. Melatonin reverses the mesenchymal phenotype through coordinated regulation of multiple EMT-associated transcription factors. In breast cancer models, melatonin downregulates Snail, Slug, and Twist1, increases E-cadherin expression, and reduces N-cadherin and vimentin expression, thereby reversing the mesenchymal phenotype (44). Mechanistically, melatonin inhibits Akt phosphorylation, reduces the inhibitory phosphorylation of GSK3β, promotes β-catenin degradation, and consequently suppresses EMT (42). Melatonin also suppresses EMT through modulation of the DJ-1/KLF17/ID-1 signaling pathway, leading to increased E-cadherin expression and reduced Snail activity (45). Consistent with these findings, melatonin reduced the CD44^+^/CD24^-^ stem-like mammosphere population and suppressed invasive capacity in both canine (CMT-U229) and human (MCF-7) mammosphere models, collectively indicating that melatonin suppresses EMT, cancer stemness, and tumor-cell plasticity (**Figure 3B**) (44).

### 5.3 Circadian regulation of tumor cell dissemination

A pivotal study by Diamantopoulou et al. demonstrated that CTC release in breast cancer is temporally regulated rather than continuous. Paired blood samples collected from patients at 04:00 and 10:00 showed that the majority of single CTCs, CTC clusters, and CTC–white blood cell clusters were detected during the rest phase, a finding reproduced across several mouse breast cancer models. Rest-phase CTCs exhibited markedly greater metastatic competence following experimental transplantation and were enriched for mitotic, cell-division, and translation-related gene programs. The temporal increase in CTC abundance primarily reflected enhanced intravasation rather than reduced circulatory clearance. CTCs also expressed receptors for circadian-regulated systemic signals, including glucocorticoids, androgens, and insulin, supporting a role for host endocrine rhythms in controlling tumor-cell dissemination. Notably, melatonin administration increased CTC generation in mice, whereas co-administration of the melatonin receptor antagonist luzindole attenuated this effect, indicating that melatonin contributes to the temporal regulation of CTC intravasation in this experimental context rather than acting as a direct promoter of metastasis. Furthermore, because breast cancer-derived CTCs showed limited evidence of an autonomous functional circadian clock, their rhythmic release appears to be driven predominantly by host circadian and endocrine cues rather than tumor cell-intrinsic clock machinery (3).

These findings are further supported by experimental studies showing that chronic circadian disruption accelerates metastatic progression by remodeling the tumor microenvironment, enhancing cancer stemness, and promoting dissemination, highlighting the importance of host circadian integrity in restraining breast cancer metastasis (46). Importantly, increased CTC release should not be interpreted as synonymous with enhanced metastatic progression. Metastasis is a multistep biological process involving tumor-cell intravasation, survival in the circulation, metastatic competence, extravasation, and successful colonization of distant organs. Although melatonin administration increased CTC generation under specific circadian conditions, extensive evidence discussed throughout this review demonstrates that melatonin concurrently suppresses EMT, cancer stemness, metabolic reprogramming, and tumor–microenvironment interactions that are critical for metastatic seeding and colonization. These observations suggest that melatonin primarily regulates the temporal dynamics of tumor-cell dissemination rather than functioning as a general promoter of metastasis. Collectively, current evidence suggests that the influence of melatonin on breast cancer metastasis is highly context-dependent and should be interpreted according to the specific stage of the metastatic cascade being examined. While numerous experimental studies demonstrate that melatonin suppresses epithelial–mesenchymal transition, local invasion, and tumor growth, its effects on CTC release appear to depend on treatment timing, host circadian phase, and the surrounding endocrine milieu, emphasizing the importance of chronobiology when considering melatonin as an adjuvant therapeutic strategy (**Figure 3C**) (3, 47). The apparent paradox may also be influenced by tumor subtype and melatonin exposure, although these possibilities have not been systematically investigated in the context of circadian CTC dissemination. In particular, physiological nocturnal melatonin signaling and pharmacological melatonin administration may not be biologically equivalent, and potential subtype- and dose-dependent effects require further investigation. Future studies should therefore distinguish circadian regulation of CTC release from subsequent metastatic seeding and colonization when evaluating the therapeutic potential of melatonin.

## 6 The local milieu: melatonin as an orchestrator of the tumor microenvironment

### 6.1 Melatonin-mediated immune remodeling of the tumor microenvironment

Melatonin exerts broad immunomodulatory effects that may contribute to a tumor microenvironment less permissive for metastatic progression. Available evidence suggests that melatonin can enhance antitumor cytotoxic responses involving natural killer cells and cytotoxic T lymphocytes while attenuating regulatory T-cell activity and immunosuppressive macrophage phenotypes. However, these conclusions are derived largely from heterogeneous experimental systems and have not been systematically validated in metastatic breast cancer (48).

More direct cancer-model evidence supports an effect of melatonin on immune-checkpoint signaling. In KRAS-mutant non-small-cell lung cancer, melatonin suppressed the YAP–PD-L1 axis, reduced tumor-cell PD-L1 expression, and shifted the tumor microenvironment toward enhanced antitumor immunity (49). In head and neck squamous-cell carcinoma, melatonin similarly inhibited EMT and PD-L1 expression through the ERK1/2–FOSL1 pathway, with associated enhancement of antitumor immune responses (50). More recently, an original hepatocellular carcinoma study showed that melatonin reduced PD-L1 expression, increased T-lymphocyte activity, and suppressed tumor growth, migration, and invasion, potentially through HIF-1α and MAPK-related signaling (51). Melatonin also modifies tumor–macrophage communication. In gastric cancer, melatonin altered the composition of tumor-derived extracellular vesicles, reduced macrophage PD-L1 expression, and restored macrophage antitumor activity (52). Earlier work in hepatocellular carcinoma likewise showed that exosomes released from melatonin-treated tumor cells altered macrophage immunosuppression through STAT3-related signaling (53).Together, these findings suggest that melatonin may suppress metastatic progression by strengthening cytotoxic immune surveillance and limiting PD-L1- and macrophage-associated immunosuppression (**Figure 3D**). Nevertheless, direct evidence defining its effects on NK cells, CD8^+^ T cells, Tregs, MDSCs, and TAMs specifically within metastatic breast cancer remains limited.

### 6.2 Melatonin-mediated metabolic reprogramming and the Warburg effect

Melatonin modulates cancer-cell metabolism and can suppress aerobic glycolysis. In tissue-isolated MCF-7 human breast cancer xenografts, physiological nocturnal melatonin reduced tumor glucose uptake and lactate production through MT1-dependent inhibition of cAMP signaling, linoleic acid uptake, and 13-HODE formation, accompanied by attenuation of AKT, c-MYC, and HIF-1α-associated metabolic signaling and reduced tumor growth (33).

Melatonin has also been proposed to redirect pyruvate away from lactate production and toward mitochondrial oxidation by limiting HIF-1α–PDK signaling and restoring pyruvate dehydrogenase activity; however, this mechanism remains largely hypothesis-driven and has not been directly validated in breast cancer models (54). More recent original studies in other tumor types further demonstrated that melatonin suppresses aerobic glycolysis by activating the SIRT3–PDH axis, thereby promoting mitochondrial pyruvate oxidation and reducing glucose consumption and lactate production (55). Because aerobic glycolysis and lactate accumulation promote EMT, immune suppression, extracellular-matrix remodeling, and metastatic niche formation, inhibition of the Warburg phenotype may contribute to the anti-metastatic effects of melatonin (**Figure 3D**). Nevertheless, direct evidence demonstrating that melatonin-mediated metabolic reprogramming suppresses breast cancer metastasis remains limited.

### 6.3 Melatonin-mediated adipocyte–tumor crosstalk

Adipose tissue is a major component of the breast tumor microenvironment and actively contributes to breast cancer progression through reciprocal communication between adipocytes and tumor cells (56). In coculture models, breast cancer cells inhibit adipocyte differentiation and establish a desmoplastic epithelial–adipose stromal microenvironment characterized by increased production of anti-adipogenic cytokines, including TNF-α, IL-6, and IL-11 (57). Melatonin disrupts this reciprocal crosstalk by restoring adipogenic differentiation through upregulation of PPARγ and C/EBPα and by suppressing the associated inflammatory cytokine response (57, 58). Melatonin also suppresses aromatase regulatory pathways and reduces local estrogen-producing capacity in breast cancer cells and adipose stromal fibroblasts (30, 31). These findings indicate that melatonin interferes with epithelial–adipose stromal communication within the breast tumor microenvironment (**Figure 3D**). However, whether melatonin directly regulates the metabolic phenotype of cancer-associated adipocytes—including lipolysis, fatty-acid transfer, adipokine secretion, and extracellular-vesicle-mediated communication—remains largely unknown and represents an important direction for future investigation.

## 7 Organ-specific metastatic niches: interaction with the circadian–melatonin axis

Metastasis is not random. Luminal breast cancers preferentially colonize bone; triple-negative and basal-like tumors metastasize to lung and liver; HER2-enriched disease disproportionately spreads to brain. Each niche imposes distinct metabolic, immune, and mechanical constraints, and each intersects with the circadian–melatonin axis in distinct ways. Following dissemination into the circulation, metastatic breast cancer cells encounter organ-specific microenvironments that determine whether disseminated tumor cells survive, enter dormancy, or successfully establish secondary tumors. Increasing evidence suggests that circadian signaling and melatonin may influence these processes in a tissue-specific manner. The strength of evidence linking melatonin to organ-specific metastatic niches varies considerably among metastatic sites. While direct experimental evidence is available for certain mechanisms, particularly in bone metastasis, other proposed interactions are primarily inferred from studies in different cancer types or non-cancer disease models and therefore require further validation in breast cancer.

### 7.1 Bone metastasis: osteoclastogenesis and bone remodeling

**Figure 4:**
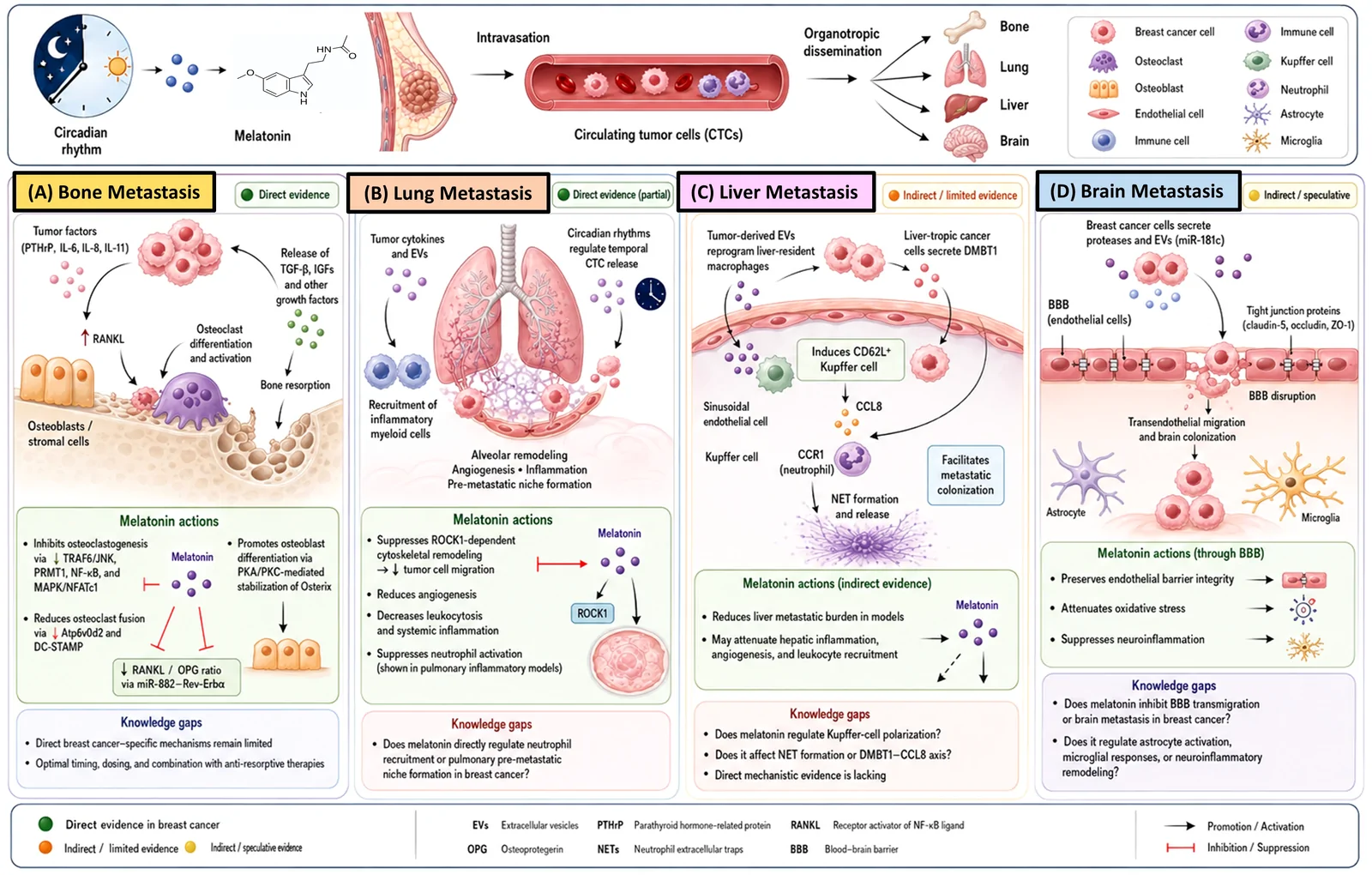
Potential roles of the circadian–melatonin axis in organ-specific metastatic niches in breast cancer. **(A)** Melatonin may suppress osteolytic bone metastasis by inhibiting osteoclastogenesis and promoting osteoblast differentiation. Available evidence supports a potential protective role of melatonin in the bone metastatic niche, although direct breast cancer-specific evidence remains limited. **(B)** In the lung, circadian rhythms and melatonin influence pulmonary metastatic niche formation through regulation of tumor-cell dissemination, inflammation, and immune responses, although the underlying mechanisms remain incompletely understood. **(C)** In the liver, melatonin may modulate hepatic metastasis by affecting Kupffer cells, inflammatory signaling, and metastatic niche formation; however, these mechanisms are currently inferred from indirect evidence, and direct validation in breast cancer is lacking. **(D)** In the brain, melatonin preserves blood–brain barrier integrity and suppresses neuroinflammation in experimental models, but whether these effects prevent breast cancer brain metastasis remains unknown. Collectively, current evidence suggests that the circadian–melatonin axis may influence organ-specific metastatic niches through distinct and context-dependent mechanisms; however, the strength of evidence varies considerably among metastatic sites, and several proposed mechanisms remain to be directly validated in breast cancer.

Bone is the most frequent site of distant metastasis in luminal breast cancer. Because osteolytic bone destruction is a hallmark of breast cancer bone metastasis, modulation of osteoclast activity represents an attractive therapeutic target for limiting metastatic progression. Bone colonization is sustained by a vicious cycle in which tumor-derived factors, including PTHrP, IL-6, IL-8, and IL-11, stimulate osteoblast-lineage and stromal cells to increase RANKL expression, thereby promoting osteoclast differentiation and bone resorption. The subsequent release of matrix-bound growth factors, including TGF-β and IGFs, further supports tumor growth within the bone microenvironment. Melatonin may interrupt this osteolytic cycle at multiple levels. It directly suppresses RANKL-induced osteoclastogenesis by inhibiting TRAF6/JNK, PRMT1, and NF-κB signaling (59). Additional evidence indicates that melatonin impairs osteoclast differentiation and fusion through inhibition of MAPK/NFATc1 signaling and downregulation of Atp6v0d2 and DC-STAMP (60). In parallel, melatonin promotes osteoblast differentiation by increasing Osterix expression and protein stability through PKA/PKC-dependent signaling (61). Melatonin also reduces the osteoblastic RANKL/OPG ratio through the miR-882/Rev-Erbα axis, thereby indirectly suppressing osteoclastogenesis (62). Experimental studies in lung and prostate cancer bone metastasis models further demonstrated that melatonin suppresses tumor-derived RANKL expression, reduces TRAP-positive osteoclast numbers, and attenuates tumor burden and osteolytic lesions. These observations suggest that similar mechanisms may operate in breast cancer bone metastasis, although direct breast cancer-specific evidence remains limited (63). Collectively, current evidence supports a potential protective role of melatonin in the bone metastatic niche, although several proposed mechanisms are extrapolated from other tumor models and await direct validation in breast cancer (**Figure 4A**).

### 7.2 Lung metastasis: circadian–melatonin regulation of the pulmonary metastatic niche

Triple-negative breast cancer exhibits a marked propensity for lung metastasis. Formation of the pulmonary pre-metastatic niche is facilitated by tumor-derived cytokines and extracellular vesicles that recruit myeloid cells and remodel the alveolar microenvironment. In addition, GALNT14 promotes pulmonary colonization by enhancing tumor-cell self-renewal within the lung niche (64). Emerging evidence indicates that metastatic dissemination is regulated by host circadian rhythms and systemic endocrine cues, with the majority of metastasis-competent circulating tumor cells being released during the rest phase (3). Because the lung represents one of the earliest vascular beds encountered by circulating tumor cells, circadian regulation of CTC release may influence pulmonary colonization, although this possibility has not yet been directly demonstrated. In breast cancer models, melatonin reduces lung metastasis partly through downregulation of ROCK1, thereby impairing cytoskeletal remodeling and tumor-cell migration (65). In addition to its direct effects on tumor-cell motility, melatonin may also modulate the pulmonary immune microenvironment. Although direct evidence in breast cancer remains limited, melatonin suppresses neutrophil activation in experimental models of pulmonary inflammation, suggesting a potential mechanism by which the circadian–melatonin axis may restrain formation of the pulmonary pre-metastatic niche. Consistent with this concept, melatonin significantly reduced lung and liver metastases, primary tumor growth, angiogenesis, and tumor-associated leukocytosis in orthotopic mouse models established with 4TLM cells derived from 4T1 murine breast carcinoma, indicating that suppression of systemic inflammatory responses contributes to its anti-metastatic activity (**Figure 4B**) (66). Nevertheless, whether melatonin directly regulates neutrophil recruitment or the pulmonary pre-metastatic niche during breast cancer lung metastasis remains to be established. Although evidence supports anti-metastatic effects of melatonin in breast cancer lung metastasis, its direct role in regulating pulmonary pre-metastatic niche formation remains incompletely understood.

### 7.3 Liver metastasis: potential influence of the circadian–melatonin axis on the hepatic metastatic niche

The liver is a unique metabolic and immunological organ that imposes substantial selective pressure on disseminated tumor cells. Successful hepatic colonization depends on dynamic interactions between metastatic tumor cells and the hepatic microenvironment, including hepatocytes, hepatic sinusoidal endothelial cells, and Kupffer cells (67). Tumor-derived extracellular vesicles contribute to the establishment of the hepatic pre-metastatic niche through interactions with liver-resident cells, particularly Kupffer cells (68). Recent evidence further demonstrated that liver-tropic breast cancer cells secrete DMBT1, which induces a distinct CD62L^+^ Kupffer-cell subset. These Kupffer cells promote neutrophil extracellular trap (NET) formation through the CCL8–CCR1 signaling axis, thereby facilitating liver metastasis (69). Although direct mechanistic studies linking melatonin to this pathway remain unavailable, melatonin significantly reduced hepatic metastatic burden together with primary tumor growth, angiogenesis, and tumor-associated leukocytosis in an orthotopic mouse model established with 4TLM cells derived from 4T1 murine breast carcinoma (66). Whether melatonin directly regulates the DMBT1–MUC1–NF-κB–CCL8 signaling cascade, CD62L^+^ Kupffer-cell differentiation, or neutrophil NETosis during breast cancer liver metastasis remains unknown (**Figure 4C**). Therefore, the proposed role of melatonin in regulating the hepatic metastatic niche currently remains hypothetical and requires direct validation in breast cancer models.

### 7.4 Brain metastasis and the circadian–melatonin axis

Brain metastasis is a clinically devastating manifestation of breast cancer and is generally associated with poor survival, particularly in patients with triple-negative or HER2-positive disease (70). Successful colonization of the central nervous system requires disseminated tumor cells to traverse the blood–brain barrier (BBB), a highly specialized endothelial interface maintained by tight-junction proteins, including claudin-5, occludin, and ZO-1 (71). Breast cancer cells disrupt BBB integrity through protease-mediated tight-junction remodeling and the secretion of extracellular vesicles carrying miR-181c, which alters endothelial actin organization and facilitates transendothelial migration (72, 73). Melatonin readily crosses brain barriers and is an important regulator of central circadian homeostasis. Evidence from neurological disease and injury models indicates that melatonin preserves BBB integrity, limits oxidative stress, and attenuates neuroinflammation (74, 75). These properties may help maintain the brain microenvironment; however, their relevance to the prevention or treatment of breast cancer brain metastasis has not been directly established. Whether melatonin regulates tumor-cell transmigration across the BBB, astrocyte–tumor communication, microglial activation, or neuroinflammatory remodeling during breast cancer brain metastasis remains unknown (**Figure 4D**). Accordingly, current evidence supports a neuroprotective role of melatonin, whereas its direct involvement in breast cancer brain metastatic colonization remains speculative.

## 8 Clinical implications

### 8.1 Circadian chronotherapy

Circadian chronotherapy, defined as the alignment of treatment administration with the patient’s endogenous circadian rhythm, has emerged as a promising strategy to optimize therapeutic efficacy while minimizing treatment-related toxicity. In breast cancer, clinical evidence remains limited compared with other malignancies. The recently published REaCT-CHRONO pragmatic multicenter randomized trial evaluated morning versus evening administration of adjuvant endocrine therapy in patients with early-stage breast cancer and assessed treatment tolerability, adherence, and patient-reported outcomes, although oncologic efficacy was not the primary endpoint (76). Clinical studies suggest that the timing of breast radiotherapy may affect normal-tissue toxicity. Morning treatment was associated with less or later-onset acute skin toxicity in observational studies, whereas the risk of late toxicity appears to depend on interactions between treatment time and individual circadian genotypes (77–80). Consequently, circadian chronotherapy has not yet been incorporated into routine breast cancer management because convincing evidence demonstrating improvements in recurrence or survival remains lacking.

### 8.2 Melatonin as an adjuvant therapy

Melatonin has attracted interest as a potential adjuvant therapy because of its generally favorable short-term safety profile and its proposed ability to enhance treatment responses or mitigate the adverse effects of conventional anticancer therapies. Earlier meta-analyses of randomized trials in patients with heterogeneous solid tumors reported lower one-year mortality, improved tumor remission or response, and reductions in several hematologic, neurologic, and constitutional toxicities when melatonin was added to chemotherapy or radiotherapy (81, 82). However, these findings should be interpreted cautiously because the contributing trials were generally small, frequently open-label, included heterogeneous advanced malignancies, and were disproportionately conducted by a limited number of research groups. Moreover, an updated Cochrane review concluded that the available evidence for the effects of melatonin on quality of life, sleep, treatment-related toxicity, tumor response, and survival in patients with cancer remains of very low certainty (83). A breast cancer–focused systematic review similarly suggested that melatonin combined with standard chemotherapy may improve quality of life and mitigate selected treatment-related adverse effects, and noted possible increases in partial response and one-year survival with doses of approximately 20 mg/day. Nevertheless, these conclusions were based on a small and methodologically limited clinical evidence base and require confirmation in adequately powered multicenter trials (84).

Preclinical studies indicate that melatonin may enhance the response of breast cancer cells to ionizing radiation by suppressing estrogen biosynthesis and proliferative signaling and by modulating oxidative stress, DNA-damage responses, and apoptosis (85). However, these laboratory findings have not yet translated into demonstrated improvements in local tumor control, recurrence, or survival. Clinical evidence regarding symptom control during breast radiotherapy is also inconsistent. A double-blind, placebo-controlled phase III trial, which was stopped early for futility, found that 20 mg nightly melatonin did not prevent or significantly improve fatigue or other patient-reported symptoms during adjuvant radiotherapy for early-stage breast cancer (86). In contrast, a subsequent single-center, triple-blind randomized placebo-controlled trial reported lower fatigue, anxiety, and depression scores among patients receiving 20 mg/day melatonin during adjuvant radiotherapy (87). These discordant findings underscore the need for adequately powered multicenter trials with standardized dosing, administration timing, circadian-phase assessment, and both patient-reported and oncologic endpoints.

### 8.3 Precision medicine and circadian-guided therapy

The integration of circadian biology into precision breast cancer therapy may require consideration of both tumor-intrinsic and patient-specific circadian characteristics. Breast cancer molecular subtype and melatonin receptor expression may represent potential stratification factors, as MT1 expression varies according to hormone receptor status (27). Tumor circadian characteristics may provide an additional layer of biological stratification, as heterogeneity in global circadian rhythm strength in luminal A tumors has been associated with EMT pathway rhythmicity, metastatic potential, and clinical outcome (13). Patient-specific circadian biomarkers may further facilitate individualized treatment scheduling. In breast radiotherapy, circadian genetic variants have been associated with time-of-day differences in treatment toxicity, supporting the potential for biomarker-guided treatment scheduling (80). Together with emerging clinical evidence on treatment timing in endocrine therapy (76, 88), these findings support a precision-medicine framework integrating tumor subtype, melatonin receptor expression, circadian biomarkers, and individualized treatment timing rather than applying uniform clock-time schedules. However, prospective validation is required before circadian-guided strategies can be routinely implemented in breast cancer care.

## 9 Knowledge gaps and future directions

Despite substantial mechanistic progress, major gaps continue to limit the clinical translation of melatonin in breast cancer metastasis. Its effects may depend on circadian phase, metastatic stage, organ-specific microenvironment, and host metabolic status rather than representing a uniformly anti-metastatic response. In particular, the observation that circulating tumor-cell release increases during the rest phase, when melatonin levels are highest, highlights the need for phase-controlled studies that distinguish effects on intravasation, metastatic seeding, and subsequent outgrowth. Most preclinical studies have used early-intervention models, leaving the effects of melatonin on established metastases poorly defined. Reliable biomarkers of individual and tumor circadian phase also require prospective validation. Moreover, interactions between melatonin and contemporary systemic treatments, including CDK4/6 inhibitors, PARP inhibitors, immune checkpoint inhibitors, and antibody–drug conjugates, remain largely unexplored. Future trials should therefore incorporate chronotype or circadian-phase assessment, standardized dosing and timing, and clinically meaningful endpoints such as metastatic recurrence, disease-free survival, and organ-specific progression.

## 10 Conclusion

The circadian–melatonin axis is increasingly recognized as an important biological framework that may influence breast cancer metastasis through endocrine signaling, tumor-cell plasticity, immune responses, metabolic remodeling, and interactions within the metastatic microenvironment. Emerging evidence further suggests that obesity-associated adipose dysfunction and adipocyte–tumor crosstalk may represent important components of this regulatory network. While accumulating experimental evidence highlights the therapeutic potential of targeting the circadian–melatonin axis, important mechanistic and clinical questions remain unresolved. Addressing these challenges through chronobiology-informed translational and clinical studies will be essential for developing more effective strategies to prevent and treat breast cancer metastasis.
